# Pulmonary Lobectomy for Early-Stage Lung Cancer with Uniportal versus Three-Portal Video-Assisted Thoracic Surgery: Results from a Single-Centre Randomized Clinical Trial

**DOI:** 10.3390/jcm12227167

**Published:** 2023-11-18

**Authors:** Davide Tosi, Alessandra Mazzucco, Valeria Musso, Gianluca Bonitta, Lorenzo Rosso, Paolo Mendogni, Ilaria Righi, Rosaria Carrinola, Francesco Damarco, Alessandro Palleschi

**Affiliations:** 1 Thoracic Surgery and Lung Transplantation Unit, Fondazione IRCCS Ca’ Granda—Ospedale Maggiore Policlinico, 20122 Milan, Italy; davide.tosi@policlinico.mi.it (D.T.); alessandra.mazzucco@asst-santipaolocarlo.it (A.M.); gianluca.bonitta@unimi.it (G.B.); lorenzo.rosso@unimi.it (L.R.); paolo.mendogni@policlinico.mi.it (P.M.); ilaria.righi@policlinico.mi.it (I.R.); rosaria.carrinola@policlinico.mi.it (R.C.); francesco.damarco@policlinico.mi.it (F.D.); alessandro.palleschi@unimi.it (A.P.); 2 Department of Patho-Physiology and Transplantation, University of Milan, 20122 Milan, Italy

**Keywords:** VATS lobectomy, uniportal, three-portal, postoperative pain, postoperative complications, minimally invasive

## Abstract

Video-assisted thoracic surgery (VATS) is a consolidated approach; however, there is no consensus on the number of ports leading to less postoperative pain. We compared early postoperative pain after uniportal and three-portal VATS lobectomy for early-stage NSCLC. In this randomized clinical trial, patients undergoing VATS lobectomy were randomly assigned to receive uniportal (U-VATS Group) or three-portal (T-VATS Group) VATS. The inclusion criteria were age ≤ 80 years and ASA < 4. The exclusion criteria were clinical T3, previous thoracic surgery, induction therapy, chest radiotherapy, connective tissue or vascular diseases, major organ failure, and analgesics or corticosteroids use. The postoperative analgesia protocol was based on NRS. Pain was measured as analgesic consumption; the secondary endpoints were intra- and postoperative complications, conversion rate, surgical time, dissected lymph nodes, hospital stay, and respiratory function. Out of 302 eligible patients, 120 were included; demographics were distributed homogeneously. The mean cumulative morphine consumption (CMC) in the U-VATS Group after 7 days was lower than in the T-VATS Group (77.4 mg vs. 90.1 mg, *p* = 0.003). Intraoperative variables and postoperative complications were comparable. The 30-day intercostal neuralgia rate was lower in the U-VATS Group, without reaching statistical significance. Patients undergoing U-VATS showed a lower analgesic consumption compared with the T-VATS Group; analgesic consumption was moderate in both groups.

## 1. Introduction

Lung cancer is the second most frequent tumour and the leading cause of cancer death [1]. Pulmonary lobectomy remains the gold standard for early-stage non-small cell lung cancer (NSCLC). The video-assisted thoracic surgery (VATS) approach had already been recommended over open surgery in 2013 [2,3], and today its advantages in terms of a shorter length of hospital stay, fewer postoperative adverse events, and less pain are well established [4,5,6,7,8]. The advantages of VATS lobectomy over the open technique were also recently confirmed by Lim et al. [9]. Different approaches for VATS lobectomy have been described, specifically based on the number of ports. We reported the three-portal (T-VATS) anterior approach described by Hansen [10] as the most commonly used approach in Italy [11]. On the other hand, the uniportal VATS pulmonary lobectomy (U-VATS) has been increasingly adopted worldwide after the standardization of the technique by Rivas [12,13]. Nonetheless, whether U-VATS is superior to T-VATS is still under debate [14].

In the early and late postoperative period after thoracic surgery, pain is certainly a relevant aspect, per sè, due to the associated complications and those related to analgesic treatment [15]. Single-port thoracoscopic surgery has been associated with less postoperative pain compared with the multi-ports (M-VATS) technique, but evidence on postoperative pain after pulmonary lobectomy is limited [16]. The only randomized controlled trial did not find statistically significant differences between U-VATS and M-VATS lobectomy in terms of postoperative pain [17]. In a recent meta-analysis comparing U-VATS with M-VATS, a pooled analysis demonstrated significantly lower pain within the first 30 days after surgery, but the use of analgesics was only reported by two studies [18].

Therefore, our group launched a randomized clinical trial in 2017. Here, we present the results of the study comparing the U-VATS and T-VATS approaches in terms of postoperative pain after pulmonary lobectomy.

## 2. Materials and Methods

### 2.1. Study Design

**Figure 1:**
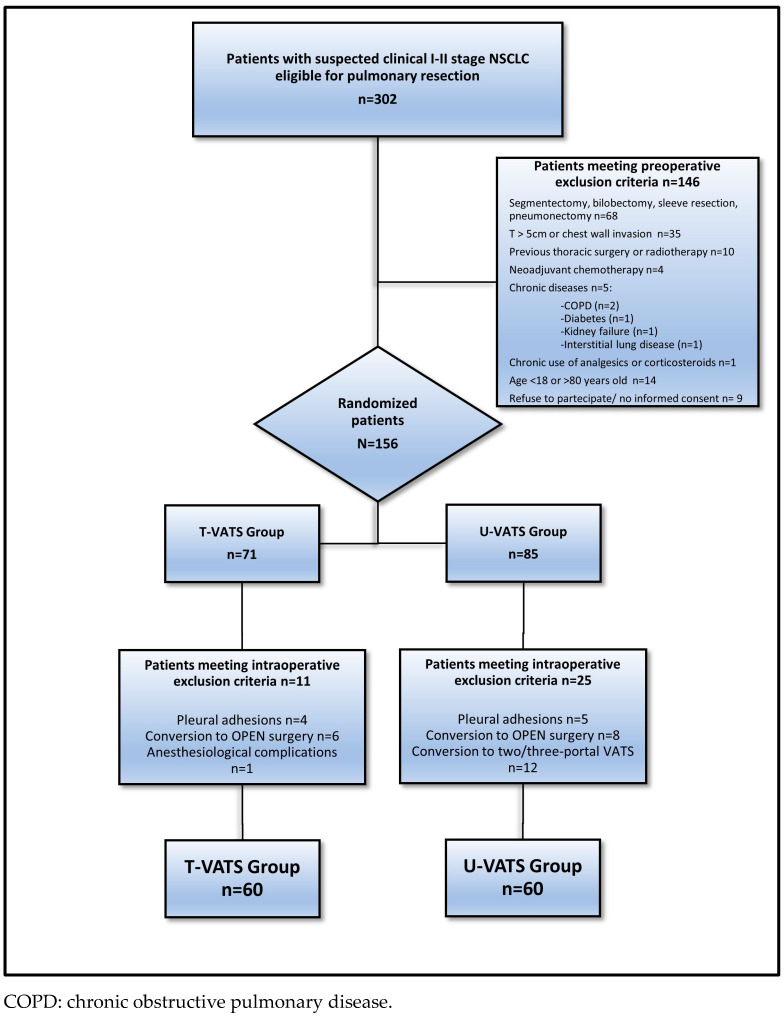
Study population.

We conducted a single-centre prospective randomized, single-blind, controlled trial (ClinicalTrials.gov: NCT03240250—study start date 3 December 2017) to compare the outcomes after pulmonary lobectomy performed with U-VATS versus T-VATS. In order to minimize imbalance with respect to the baseline variables, we used a minimization algorithm to randomize patients consecutively as they were enrolled [19]; in brief, minimization is considered to be a dynamic method since the randomization list is not produced before the trial start but during participant recruitment. All subjects gave their informed consent for inclusion before they participated in this study. This study was conducted in accordance with the Declaration of Helsinki, and the protocol was approved by the Ethics Committee of Fondazione IRCCS Ca’ Granda Ospedale Maggiore Policlinico, Milan, Italy, on 21 February 2017 (reference number 86_2017). Patients underwent routine preoperative exams, including a computed tomography (CT) scan, a total body positron emission tomography (PET) scan, and pulmonary function tests (PFTs). The preoperative evaluation was performed according to the European Society of Anesthesiology guidelines [20]. Demographic and clinical pre-, intra-, and postoperative variables were prospectively collected in a dedicated database. In particular, the following variables were recorded: age, level of education, sex, body mass index (BMI), Thoracoscore [21], preoperative PFTs, Charlson Comorbidity Index [22], Karnofsky Performance Status [23], ASA (American Society of Anaesthesiology) scale [24], lobectomy site, presence of pleural adhesions, surgical time, air leakage at the end of surgery, number of harvested lymph nodes, number of sampled nodal stations, Numerical Rating Scale (NRS), cumulative morphine consumption (CMC), duration of postoperative air leaks, chest drain dwelling time, postoperative complications, 30 day-mortality, and 30-day intercostal neuralgia. Intercostal neuralgia was assessed clinically and defined as a referred pain or burning sensation, continuous or intermittent, in the intercostal space at the site of surgery. Postoperative complications were categorized as follows: surgical site infection; prolonged air leaks (>7 days); pulmonary complications (fever and/or high inflammatory markers associated with pulmonary consolidation at chest X-ray requiring antimicrobial therapy, pulmonary atelectasis requiring bronchial aspiration, lung hernia, pneumothorax requiring chest drain placement); cardiac complications; and other complications. In the operating theatre, right before surgery, patients were randomly assigned to either the T-VATS Group, and therefore underwent three-portal VATS lobectomy and lymphadenectomy, or the U-VATS Group, and therefore underwent uni-portal VATS lobectomy and lymphadenectomy [25] (Figure 1). Surgeons in charge of performing the planned surgery had already completed their learning curve for both techniques. The study followed the Consolidated Standards of Reporting Trials (CONSORT) reporting guideline for intervention trials [26].

### 2.2. Study Endpoints

The primary endpoint was postoperative pain, measured as analgesic consumption. The secondary endpoints were the rate of intra- and postoperative complications and the respiratory functional outcome in the early period.

### 2.3. Inclusion and Exclusion Criteria

All patients referred to our Department from March 2017 to October 2020 were screened: patients with clinical stage I-II NSCLC scheduled for a VATS pulmonary lobectomy were included in this study and enrolled after signing a specific informed consent.

Preoperative exclusion criteria were:

- Age < 18 or >80 years;
- Clinical stage > II;
- Clinical T3 disease;
- ASA score > 3;
- Patients evaluated for sublobar resection, bilobectomy, sleeve lobectomy, or pneumonectomy;
- Previous thoracic surgery, induction therapy, or chest radiotherapy
- Connective tissue diseases, peripheral vascular diseases, major organ failure (kidney, liver, heart);
- Severe chronic obstructive pulmonary disease (COPD), asthma, or interstitial lung disease;
- Chronic use of analgesics or corticosteroids;
- Lack of informed consent.

Intraoperative exclusion criteria were:

- Massive pleural adhesions;
- Conversion to open surgery or supplementary trocar.

### 2.4. Surgical Techniques

For both surgical approaches, the same protocol was applied regarding anaesthesia and positioning. General anaesthesia and a double-lumen endobronchial tube were used; patients were positioned in lateral decubitus, with the bed flexed to widen intercostal spaces. Patients were monitored in a standard fashion. In the T-VATS Group patients, a 4 to 5 cm muscle-sparing mini-thoracotomy was performed through the 4th or 5th intercostal space; two thoracoscopy ports were placed in the 7th or 8th intercostal space. After pulmonary lobectomy and lymphadenectomy, a chest tube was inserted through the lower anterior intercostal incision [10]. In the U-VATS Group patients, a single 4 to 5 cm muscle-sparing mini-thoracotomy was performed through the 4th or 5th intercostal space, with no further incisions. After pulmonary lobectomy and lymphadenectomy, a chest tube was inserted through the incision [12]. In both groups, a soft tissue retractor (Alexis Retractor, Applied Medical, Rancho Santa Margarita, CA, USA) was placed in the mini-thoracotomy. A systematic lymphadenectomy was routinely performed [27].

### 2.5. Postoperative Care

Patients were treated according to our centre’s standard of care, including short-term antimicrobial prophylaxis, venous thromboembolic prophylaxis, early mobilization, and respiratory physiotherapy carried out with the support of a dedicated team of physiotherapists. All patients enrolled in this study were extubated in the operating theatre. Seven patients were transferred to the intensive care unit for monitoring on the first postoperative day (four in the U-VATS Group and three in the T-VATS Group). The chest tube was removed when both a daily output of less than 300 mL/day and no air leaks were observed.

### 2.6. Pain Evaluation and Analgesic Protocol

For pain intensity evaluation, the NRS was used: 0 identifies the total absence of pain, while 10 is the worst imaginable pain. Pain was rated and registered every four hours after surgery; mean NRS was then calculated daily from postoperative days (PODs) 1 to 7. For all patients, intravenous infusion of 1 mg/h of morphine started about one hour before the end of surgery. At the end of the procedure, surgeons performed an intercostal nerve block of 3–4 intercostal spaces under thoracoscopic vision, using 2–5 mL of ropivacaine 7.5% per intercostal space; no paravertebral catheter was placed. After surgery, postoperative analgesia was initiated in the recovery room, consisting of intravenous morphine infusion, which was maintained at 1 mg/h for 6 h, 1000 mg intravenous acetaminophen three times a day, and 30 mg intravenous ketorolac three times a day. After the first 6 h, the morphine infusion rate was reduced to 0.5 mg/h. The infusion rate was then adjusted according to NRS, which was assessed every 4 h as follows:

- NRS < 4: infusion rate was decreased by 0.125 mg/h;
- NRS = 4: infusion rate was not altered;
- NRS > 4: infusion rate was increased by 0.125 mg/h;
- NRS > 6: 1 mg bolus of morphine was administered.

Morphine infusion was stopped when an NRS lower than 4 was reported by a patient receiving a 0.125 mg/h dose or if any side effect occurred (dizziness, confusion, vertigo, nausea, or vomiting). After chest drain removal, acetaminophen (1000 mg three times a day) was administered orally, and ketorolac 30 mg was administered only with an NRS > 4. Patients were discharged with PRN pain medication only; data on analgesic consumption after discharge were also collected. Non-steroidal anti-inflammatory drug (NSAID) consumption was taken into consideration and converted to morphine equivalents according to equianalgesic charts, thus calculating the cumulative morphine consumption (CMC) [28].

### 2.7. Statistical Analysis

The sample was described using mean and standard deviation or absolute and percentage frequencies, as appropriate. The primary efficacy outcome was tested using an equality two-sided *t*-test for independent data; the normality assumption was evaluated using both the Shapiro–Wilk test and visual inspection of the Q–Q plot. To evaluate the balance between study groups, we considered both the Omnibus test of joint orthogonality [29] and a value < 0.25 for standardized mean difference (SMD) [30]. The secondary outcomes were evaluated using a statistical methodology similar to the one for the primary outcome for continuous variables. For binary outcomes, we performed the Chi-square test. The Bonferroni correction was used for multiple comparisons. All statistical tests were two-sided, and the confidence intervals were at 95%. All statistical analyses were performed using R statistical software 4.3.0 [31].

Primary and secondary outcomes were analysed according to the modified intention-to-treat principle. The modified intention-to-treat data were defined as all randomized patients, with the exception of those who were converted to different assigned treatments in the perioperative phase, were excluded and replaced with another randomized patient [32]. A supplementary analysis was performed, considering all patients who matched the inclusion criteria and underwent surgery (randomized plus converted). We calculated that a total sample of 120 participants would provide the trial with 85% power, with a two-sided alpha of 0.05, to detect a significant treatment effect of 20% in mean difference [33]. Considering the review of our medical record, we assumed a standard deviation within each study group of 18 mg and a cumulative morphine mean consumption of 50 mg for the multi-port VATS Group [34].

## 3. Results

During the study period, out of 302 patients with clinical I-II NSCLC stage eligible for pulmonary resection, 146 patients met the preoperative exclusion criteria and were therefore excluded. After randomization, 36 patients were excluded from this study (14 patients converted to open surgery, 9 patients with massive pleural adhesions, and 12 patients converted from uni- to multi-portal VATS). One patient experienced anaesthesia-related airway complications and was therefore excluded. Thus, our sample size was 120 patients (Figure 1).

Patients’ demographics and pre-operative variables were similar in the two study groups; all included patients were Caucasian. The Omnibus test of joint orthogonality did not show evidence of a lack of randomization balance across baseline characteristics between the two groups (*p* = 0.459), or the SMD, which was below 0.25 for all variables (Table 1).

The mean cumulative morphine consumption (CMC) after 7 days was 90.1 mg (SD = 26.5) in the T-VATS Group and 77.4 mg (SD = 17.9) in the U-VATS Group; this difference was statistically significant (*p* = 0.03, difference in means =15.7 mg, 95% CI: 4.45–20.82) (Table 2).

The analysis performed on the two groups including patients converted to open surgery or from uni- to multi-portal VATS also showed a difference: CMC after 7 days was 94.5 mg (SD = 23.3) in the T-VATS Group and 85.2 mg (SD = 19.3) in the U-VATS Group (*p* = 0.07, difference in means = 9.3 mg, 95% CI: 2.5–16.3). The analysis of postoperative pain measured with mean NRS did not show significant differences between the two groups; in both U-VATS and T-VATS patients, the mean NRS decreased from POD 1 to POD 7 (Table 3).

In addition to CMC, there were no other statistically significant differences in intraoperative data, postoperative data, and complications (Table 4).

Seven patients in each group were converted to open surgery. The skin-to-skin operative time was similar in the two groups: 232 min (SD = 55) and 220 min (SD = 56) in the U-VATS and T-VATS Groups, respectively (*p* = 0.251). All patients had a radical (R0) resection; the pathological stage did not statistically differ between the two groups. Mortality at 30 days was nil in both groups. The U-VATS Group had a lower rate of intercostal neuralgia at 30 days, without reaching statistical significance (*p* = 0.068). There were no readmissions due to uncontrolled pain or to analgesic-related complications. Recovery of pulmonary function was comparable between the two groups after 1 month (Table 5).

## 4. Discussion

We present the results of our prospective randomized clinical trial comparing the outcomes of patients undergoing VATS pulmonary lobectomy for early-stage NSCLC with uni- versus three-portal VATS. Our experience suggests that the U-VATS technique is associated with lower analgesic consumption in the immediate postoperative period. Even though oncological adequacy remains the main target when studying surgical approaches for cancer, postoperative pain is a key factor in determining clinical outcomes. In fact, pain following thoracic surgery could delay patients’ respiratory function recovery and affect their quality of life. The advent of minimally invasive thoracic surgery allowed for performing pulmonary resections with significant improvements in postoperative pain and complications: thus, pulmonary lobectomy via VATS has been the preferred approach for early-stage NSCLC since the early 2010s in most centres [2]. Since then, many VATS techniques have been described, and U-VATS is considered less invasive; however, to this day there are no data on the superiority of U-VATS over other techniques in terms of postoperative pain. On the one hand, by using the uniportal technique, surgeons risk damaging only one intercostal nerve; on the other hand, instrument crowding through a single incision may increase the risk of nerve injury. The few data providing evidence on this matter mostly come from retrospective studies that used NRS or VAS to evaluate postoperative pain. A recent systematic review and meta-analysis comparing uniportal and M-VATS lobectomy (the latter including two, three, and four-portal VATS) showed lower morbidity, mortality, and hospital stay in patients undergoing U-VATS [16]. Louis et al. also compared uniportal and M-VATS lobectomy in a prospective study: the authors found a lower consumption of analgesics in patients undergoing U-VATS. However, it should be noted that patients requiring conversion to M-VATS were assigned to the multiportal group [35]. The only randomized controlled trial comparing U-VATS and M-VATS lobectomy in terms of postoperative pain was published in 2016 by Perna et al. The authors did not find differences in terms of postoperative pain, measured as visual analogue pain and median morphine use. However, it should be noted that the multiportal group included patients undergoing lobectomy with both the Duke (bi-portal) and Copenhagen (three-portal) VATS approaches [17]. A recent retrospective study based on the Italian VATS Group database including patients from 49 Italian centres comparing 172 U-VATS and 1808 T-VATS lobectomies reported a higher risk of NRS > 3 on POD 2 and 3; nonetheless, the evaluation of NRS at discharge was comparable between the two groups [11]. The most important limitation of that study was that the use of analgesics was not measured. When designing our study protocol, which aimed at comparing postoperative pain after U-VATS and T-VATS lobectomy, we chose CMC to estimate pain following thoracic surgery [27]. This parameter allowed us to obtain a more objective evaluation of postoperative pain [27,36]. It could be argued that many centres are using patient-controlled analgesia (PCA); however, our opinion is that the use of this method would have influenced data on CMC, as it depends on the patient’s attitude towards pain and analgesics. Moreover, our study aimed at comparing postoperative pain after U-VATS and T-VATS lobectomy in patients receiving the same treatment under the same conditions, rather than proposing a generalizable protocol for postoperative analgesia in thoracic surgery. First, our data showed a low CMC in both groups, thus highlighting the low invasiveness of VATS (performed with both techniques) in terms of postoperative pain. Nonetheless, in light of the statistically significant difference found between the two groups in terms of CMC, it was necessary to assess the real clinical significance of this result. The Minimum Clinic Significant Difference (MCID) is a threshold value that can ensure that the effects are clinically significant [37]. As a difference in CMC of more than 40% was never detected between the two groups, we cannot assert that the clinical impact is significant. The analysis of mean CMC in the first 7 days after surgery showed that patients undergoing U-VATS required fewer analgesics: this might have been a consequence of the reduced number of intercostal spaces involved with this technique. Moreover, U-VATS does not require the use of trocars, which may cause intercostal nerve damage. The analysis of mean NRS did not show statistically significant differences between the two groups: this result was expected, as analgesics were administered based on patients’ NRS, in accordance with the pharmacologic protocol of this study. In both groups, NRS progressively decreased from POD 1 to POD 7. One month after surgery, only three patients in the U-VATS Group and nine patients in the T-VATS Group suffered from intercostal neuralgia. Even though the difference between the two groups did not reach statistical significance, it is considerable, and we could speculate that it might be the consequence of the higher number of incisions, even though this study is not powered to assess this difference. The analysis of intraoperative data did not identify significant differences between the two groups: namely, mean operating time was only 12 min higher in the U-VATS Group, despite the technical challenges of this approach. Regarding the conversion rate, 19 patients in the U-VATS Group required conversion (8 to the open technique, 12 to the multiportal VATS technique), and 6 subjects in the T-VATS Group required conversion to open surgery. These results are consistent with the complexity of the uniportal technique, particularly when dealing with more challenging surgical cases. There was no statistically significant difference in terms of chest drain dwelling time between the two groups, even though it was shorter in the U-VATS Group (4.0 days vs. 4.7 days in the T-VATS Group). Postoperative mean hospital stay was comparable between the two groups; however, one should consider that this parameter is influenced by logistics. When analysing data on lymph node dissection, the number of node stations and the total number of lymph nodes sampled were both adequate and did not differ between the U-VATS and T-VATS Group. Both techniques can be considered oncologically adequate in spite of the absence of data regarding mid- and long-term follow-up. The spirometric data obtained 7 days after surgery showed a decrease in mean forced expiratory volume in the 1st second (FEV1) and mean forced vital capacity (FVC) in both groups compared with baseline values, with a partial recovery 30 days after surgery; the data did not demonstrate statistically significant differences between the U-VATS and T-VATS Group in terms of respiratory function.

Our study has some limitations. The oncological follow-up available was limited, and we only collected data on lymph node dissection. Also, some data on patients’ postoperative respiratory function are missing. Finally, our research took place over a prolonged period of time. However, when comparing the results of patients enrolled in the first year of the study with those undergoing surgery during the last period of this study, we did not find differences in terms of postoperative pain, complications, and surgical time.

A key point of this study is the homogeneity in the two arms in relation to the surgery applied. In fact, randomization with minimization allowed us to obtain groups with patients who received exactly the planned procedure [38]. On the other hand, analysis of all selected patients (including converted patients) confirmed a difference in the use of analgesics between the two groups, although the difference was not statistically significant.

## 5. Conclusions

The results of our study show that patients undergoing U-VATS lobectomy require fewer analgesics in the immediate postoperative period than those undergoing T-VATS, given a similar NRS. However, considering MCID, this difference in analgesic consumption was not clinically significant, and low cumulative morphine consumption was found in both groups. Notably, patients in both groups only experienced mild postoperative pain, with a low prevalence of postoperative complications, thus confirming the adequacy and safety of both the U-VATS and T-VATS techniques for the treatment of early-stage NSCLC.

## Figures and Tables

**Table 1:** Patients’ demographics and preoperative characteristics.

	U-VATS Group (n = 60)	T-VATS Group (n = 60)	SMD	*p*-Value
Age, years, mean (SD)	66.7 (11.3)	68.8 (8.7)	0.21	0.26
Level of education, n (%) A B C D	5(8.3) 11 (18.3) 28 (46.7) 16 (26.7)	2 (3.4) 18 (30.0) 29 (48.3) 11 (18.3)	0.213 0.249 0.042 0.191	0.26 0.14 0.85 0.27
Male, n (%)	31 (51.7)	30 (50.0)	0.142	0.85
Thoracoscore, mean (SD)	2.0 (1.2)	2.3 (1.3)	0.242	0.19
BMI, kg/m^2^, mean (SD)	26.0 (3.9)	25.6 (4.4)	0.071	0.60
FEV1 %, mean (SD)	98.8 (16.9)	101.7 (23.9)	0.143	0.45
FVC %, mean (SD)	108.1 (23.1)	113.4 (20.5)	0.241	0.23
FEV1/FVC %, mean (SD)	74.0 (9.2)	71.8 (10.3)	0.233	0.21
DLCO %, mean (SD)	83.3 (18.4)	79.2 (15.9)	0.243	0.20
DLCO/VA %, mean (SD)	91.1 (16.1)	87.6(17.4)	0.211	0.29
Charlson Comorbidity Index, mean (SD)	4.7(1.8)	5.0 (1.5)	0.184	0.51
Karnofsky Performance Status %, mean (SD)	96.0 (5.3)	94.8 (5.4)	0.226	0.41
ASA score, n (%)				
2	38 (63.3)	31 (51.7)	0.22	0.49
3	22 (36.7)	29 (48.3)	0.22	0.20
Right upper lobe lobectomy, n (%)	20 (33.3)	25 (41.7)	0.19	0.34
Middle lobe lobectomy, n (%)	6 (10)	5 (8.3)	0.07	0.75
Right lower lobe lobectomy, n (%)	10 (16.7)	9 (15.0)	0.05	0.80
Left upper lobe lobectomy, n (%)	15 (25)	13 (21.7)	0.07	0.67
Left lower lobe lobectomy, n (%)	9 (15)	8 (13.3)	0.06	0.79

Level of education A: primary school; B: middle school; C: high school; D: degree; SMD: standardized mean difference; SD: standard deviation; BMI: body mass index; FEV1: forced expiratory volume in the first second; FVC: forced vital capacity; DLCO: diffusion lung capacity for carbon monoxide; VA: alveolar volume.

**Table 2:** Cumulative morphine consumption.

	U-VATS Group (n = 60)	T-VATS Group (n = 60)	*p*-Value	Difference in Means (CI)
CMC 1 day (mg), mean (SD)	30.4 (18.3)	37.9 (20.5)	0.037	7.5 (0.52–14.47)
CMC 2 days (mg), mean (SD)	49.8 (19.5)	62.9 (21.2)	<0.001	13.1 (5.82–20.37)
CMC 3 days (mg), mean (SD)	65.2 (16.3)	76.1 (20.2)	0.002	10.9 (4.33–17.46)
CMC 5 days (mg), mean (SD)	73.4 (16.6)	85.4 (21.6)	<0.001	12.0 (5.04–19.00)
CMC 7 days (mg), mean (SD)	77.4 (17.94)	90.1 (26.48)	0.003	12.7 (4.45–20.82)

**Table 3:** Numeric rating scale (NRS).

	U-VATS Group (n = 60)	T-VATS Group (n = 60)	*p*-Value ^a^
NRS POD 1, mean (SD)	2.3 (1.2)	2.1 (1.3)	0.99
NRS POD 2, mean (SD)	1.9 (1.1)	2.1 (1.1)	0.99
NRS POD 3, mean (SD)	1.5 (1.2)	1.5 (1.1)	0.99
NRS POD 4, mean (SD)	1.0 (1.3)	1.3 (1.3)	0.99
NRS POD 5, mean (SD)	0.8 (1.2)	1.0 (1.1)	0.99
NRS POD 6, mean (SD)	0.5 (0.9)	0.8 (1.0)	0.28
NRS POD 7, mean (SD)	0.3 (0.6)	0.5 (0.9)	0.59

^a^ *p*-value adjusted using Bonferroni correction.

**Table 4:** Intraoperative and postoperative data and complications.

	U-VATS Group (n = 60)	T-VATS Group (n = 60)	*p*-Value
Pleural adhesions, n (%)	18 (30)	21 (35)	0.56
Air leakage at the end of surgery, n (%)	6 (10)	10 (16.7)	0.29
Surgical time, minutes, mean (SD)	232 (55)	220 (56)	0.25
N° lymph nodes, mean (SD)	14.8 (6.2)	15.2 (6.6)	0.69
N° lymph node stations, mean (SD)	6.2 (1.6)	6.3 (1.0)	0.84
R0 resection, n (%)	60 (100)	60 (100)	NS
Postoperative air leaks duration, days, mean (SD)	1.2 (2.0)	1.4 (2.3)	0.49
Chest drain dwelling time, days, mean (SD)	4.0 (2.0)	4.7 (2.4)	0.06
Hospital stay, days, mean (SD)	6.3 (2.6)	6.6 (2.4)	0.47
Prolonged air leaks, n (%)	5 (8.3)	5 (8.3)	0.99
Surgical site infection, n (%)	0 (0.0)	0 (0.0)	0.99
Pulmonary infection, n (%)	4 (6.7)	4 (6.7)	0.99
Arrythmia, n (%)	3 (5.0)	2 (3.3)	0.65
Other complications, n (%)	0 (0.0)	1 (1.7)	0.31
30 days intercostal neuralgia, n (%)	3 (5.0)	9 (15.0)	0.07
30 days mortality, n (%)	0 (0.0)	0 (0.0)	0.99

**Table 5:** Postoperative pulmonary function tests 7 and 30 days after surgery.

7 POD	U-VATS Group (n = 50)	T-VATS Group (n = 46)	*p*-Value **
FEV1 %, mean (SD)	70.4 (16.1)	71.6 (16.3)	0.84
FVC %, mean (SD)	75.3 (16.2)	77.1 (17.0)	0.06
FEV1/FVC % mean (SD)	74.0 (7.2)	73.2 (8.1)	0.99
**30 POD**	**U-VATS Group (n = 50)**	**T-VATS Group (n = 39)**	***p*** -Value **
FEV1 %, mean (SD)	78.0 (17.3)	78.0 (18.1)	0.95
FVC %, mean (SD)	83.0 (17.2)	86.0 (18.0)	0.82
FEV1/FVC % mean (SD)	75.0 (6.5)	72.0 (8.9)	0.49

FEV1: forced expiratory volume in 1 s; FVC: forced vital capacity. ** *p*-value calculated with one-way ANCOVA.

## Data Availability

Data supporting this study are available from the authors upon reasonable request.
